# Hierarchical genetic structure shaped by topography in a narrow-endemic montane grasshopper

**DOI:** 10.1186/s12862-016-0663-7

**Published:** 2016-05-05

**Authors:** Víctor Noguerales, Pedro J. Cordero, Joaquín Ortego

**Affiliations:** Grupo de Investigación de la Biodiversidad Genética y Cultural, Instituto de Investigación en Recursos Cinegéticos - IREC (CSIC, UCLM, JCCM), Ronda de Toledo 12, E-13071 Ciudad Real, Spain; Department of Integrative Ecology, Estación Biológica de Doñana (EBD-CSIC), Avda. Américo Vespucio s/n, E-41092 Seville, Spain

**Keywords:** *Chorthippus saulcyi moralesi*, Ecological niche modelling, Hierarchical genetic structure, Isolation by environment, Isolation by resistance, Landscape genetics, Pyrenees, Topographic complexity

## Abstract

**Background:**

Understanding the underlying processes shaping spatial patterns of genetic structure in free-ranging organisms is a central topic in evolutionary biology. Here, we aim to disentangle the relative importance of neutral (i.e. genetic drift) and local adaptation (i.e. ecological divergence) processes in the evolution of spatial genetic structure of the Morales grasshopper (*Chorthippus saulcyi moralesi*), a narrow-endemic taxon restricted to the Central Pyrenees. More specifically, we analysed range-wide patterns of genetic structure and tested whether they were shaped by geography (isolation-by-distance, IBD), topographic complexity and present and past habitat suitability models (isolation-by-resistance, IBR), and environmental dissimilarity (isolation-by-environment, IBE).

**Results:**

Different clustering analyses revealed a deep genetic structure that was best explained by IBR based on topographic complexity. Our analyses did not reveal a significant role of IBE, a fact that may be due to low environmental variation among populations and/or consequence of other ecological factors not considered in this study are involved in local adaptation processes. IBR scenarios informed by current and past climate distribution models did not show either a significant impact on genetic differentiation after controlling for the effects of topographic complexity, which may indicate that they are not capturing well microhabitat structure in the present or the genetic signal left by dispersal routes defined by habitat corridors in the past.

**Conclusions:**

Overall, these results indicate that spatial patterns of genetic variation in our study system are primarily explained by neutral divergence and migration-drift equilibrium due to limited dispersal across abrupt reliefs, whereas environmental variation or spatial heterogeneity in habitat suitability associated with the complex topography of the region had no significant effect on genetic discontinuities after controlling for geography. Our study highlights the importance of considering a comprehensive suite of potential isolating mechanisms and analytical approaches in order to get robust inferences on the processes promoting genetic divergence of natural populations.

**Electronic supplementary material:**

The online version of this article (doi:10.1186/s12862-016-0663-7) contains supplementary material, which is available to authorized users.

## Background

Understanding the factors structuring genetic variation in natural populations is a paramount topic in evolutionary biology [1, 2]. The genetic structure of populations is primarily determined by inter-population dispersal rates and realized gene flow, which in turn are shaped by geography, environment, historical processes and, more frequently, their combined effects [3–5]. The isolation-by-distance (IBD) model predicts that genetic differentiation increases with Euclidean geographic distance because of limited dispersal and genetic drift [6, 7]. Even though this classic model explains spatial patterns of genetic differentiation in a wide range of organisms ([6], but see review in [8]), it does not consider more sophisticated information than straight-line geographic distances and assumes landscape homogeneity, an unrealistic scenario for most natural systems [9, 10]. Recently, the emergence of landscape genetics has explicitly incorporated landscape complexity into the study of evolutionary processes [9, 10], bringing new approaches that take into account the ability of organisms to disperse across different landscape features according with the resistance that they offer to movement (i.e. isolation-by-resistance, IBR [11, 12]; e.g., [13, 14]).

Beyond geography and the spatial configuration of connecting corridors and isolating barriers, environment can also play a major role in shaping spatial patterns of genetic differentiation [4, 15]. This occurs when populations inhabiting ecologically dissimilar habitats experience limited gene flow due to the low performance of immigrants arriving to areas where they may not be locally adapted or as consequence of the reluctance of individuals to cross or establish in unfamiliar habitats (i.e. isolation-by-environment, IBE [15–17]). Recent research has revealed the ubiquity of IBE patterns, highlighting the importance of ecological factors in shaping genetic structure of natural populations (see meta-analyses in [4, 18]). The contribution of environment relative to geography in explaining spatial patterns of genetic variation may vary depending on species characteristics and ecological features of the natural systems [18, 19]. Indeed, different isolating mechanisms are not mutually exclusive and gene flow is often influenced by a combination of geographical and ecological factors [5, 20, 21]. Given that geography and environment are often highly correlated, disentangling their relative impact on population genetic differentiation harbors inherent analytical difficulties (see [22] for a discussion about eco-spatial autocorrelation) that have promoted the progressive development of more robust and accurate statistical methods [23–27]. However, only a few studies have jointly considered the relative effects of geography, landscape composition and environmental heterogeneity on either landscape-level [28, 29] or range-wide patterns of genetic structure [5].

**Fig. 1:**
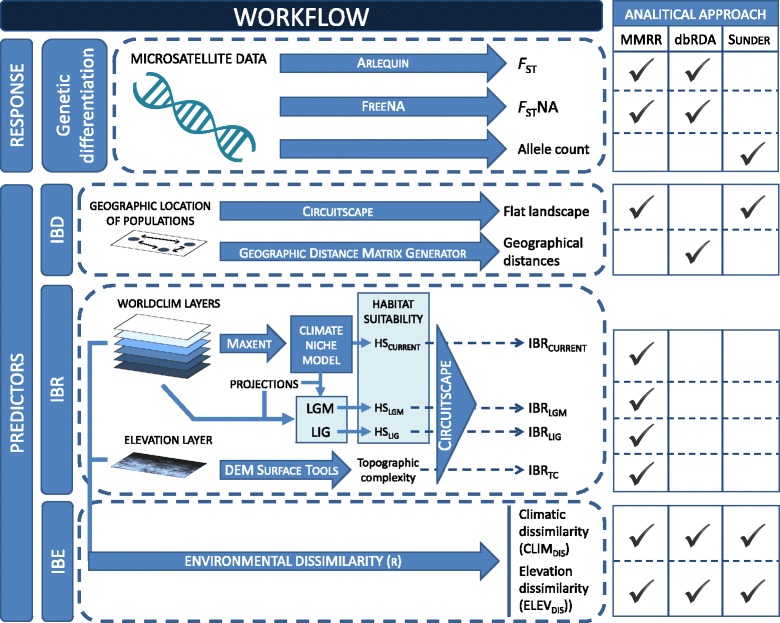
Workflow summarizing the methodological approach employed in this study to analyze the relative contribution of isolation by distance (IBD), isolation by resistance (IBR), and isolation by environment (IBE) in structuring genetic variation in the Pyrenean Morales grasshopper. The response variables and predictors considered for each analytical approach (MMRR, dbRDA, and SUNDER) are indicated. HS: Habitat suitability; LGM: Last glacial maximum; LIG: Last interglacial; MMRR: multiple matrix regression with randomization; dbRDA: distance-based redundancy analysis

The Pyrenean Morales grasshopper (*Chorthippus saulcyi moralesi*) (Orthoptera: Acrididae) is a narrow-endemic subspecies belonging to the *Chorthippus binotatus* group, exclusively distributed in central Aragón and Catalonia Pyrenean mountains (see [30], for taxonomic status and detailed description). It is a winged grasshopper primarily feeding on gramineous herbs [30] and patchily distributed across a gradient of habitats, including submediterranean shrub formations, mesophile grasslands, montane shrubby vegetation and subalpine open grasslands located at elevations ranging between 1100 and 2400 m.a.s.l. [30, 31]. Its distribution range is restricted to a narrow longitudinal axis with an east–west orientation characterized by a gradient of precipitation and temperature, from Atlantic to Mediterranean climate regimes [31]. The Pyrenees constitute the natural northern border of the Iberian Peninsula and present a high topographic and environmental complexity, rich biodiversity and considerable number of endemic species [32]. These mountains experienced dramatic climate fluctuations during the Pleistocene [33], which are expected to have strongly influenced the demographic history and altered the distribution of many organisms of the region [34]. Despite the wide variety of habitats and altitudinal ranges occupied by the Pyrenean Morales grasshopper, populations at elevations lower than 1400 m and above 2100 m are anecdotal [30, 31]. This suggests that valley bottoms and mountain tops, together with the complex topographic complexity of the region, may act as barriers to dispersal in this species [35, 36]. Thus, our study system has a great potential to examine the relative role of geography, environmental heterogeneity and present and past configuration of suitable habitats (i.e. corridors and barriers to dispersal) in shaping patterns of genetic differentiation throughout the entire distribution range of a narrowly distributed taxon [37]. We first analyzed spatial patterns of genetic structure and then employed a suite of complementary statistical approaches to test three different plausible scenarios of population differentiation (see Fig. 1 for a summary of the workflow employed in this study). In particular, we used multiple matrix regressions with randomization (MMRR, [24]), distance-based redundancy analyses (dbRDA, [38]) and geostatistical modelling based on Bayesian inference [25] to test whether the spatial pattern of genetic differentiation in the Pyrenean Morales grasshopper is explained by i) geographic distances (IBD), ii) resistance distances based on topographic complexity and current and past (last glacial maximum and last interglacial) climate suitability (IBR); and iii) altitudinal and environmental dissimilarity between populations (IBE) (Fig. 1). If geography, topography or corridors/barriers defined by habitat suitability are identified as the major drivers of genetic structure, then migration-drift equilibrium and neutral divergence can be regarded as the main evolutionary force shaping genetic discontinuities [5]. A predominant or independent significant effect of environment on disrupting gene among populations would point to a role of ecological divergence and local adaptation processes in the evolution of spatial genetic structure [39].

## Methods

### Population sampling

**Fig. 2:**
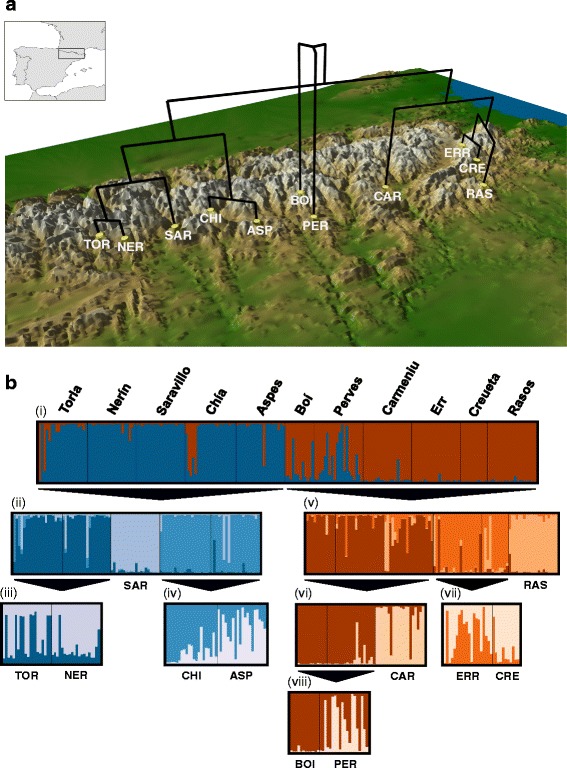
Population genetic structure in the Pyrenean Morales grasshopper. Panel **a** shows sampling sites of the species and phylogenetic relationships among the 11 populations inferred from a neighbor-joining (NJ) tree based on Cavalli-Sforza and Edwards chord distances (*D*_c_). The tree was plotted on a topographic map of the Pyrenees using the software GENGIS [ 103 ]. We downloaded topographic data from NASA Shuttle Radar Topographic Mission (SRTM Digital Elevation Data, [73]) as 90 m resolution digital elevation model and subsequently transformed to 30 arc-sec (*c*. 1 km) resolution for representation. Panel **b** represents the results of genetic assignment of 202 individuals of the Pyrenean Morales grasshopper based on the Bayesian method implemented in STRUCTURE. We performed hierarchical analyses for subsets of populations considering the most probable *K*-value inferred at the previous hierarchical level (Additional file 1; Figure S2). Each individual corresponds to a vertical bar partitioned into *K*-colored segments that represent the individual’s probability of belonging to the cluster with that color. Black lines separate individuals from different populations. Population codes as in Table 1

Between 2012 and 2014, we collected 202 individuals from 11 populations of the Pyrenean Morales grasshopper. We aimed to sample populations throughout the entire distribution range of the species (~7 000 km^2^; Fig. 2a) based on occurrence-data available in the literature [30, 31] and our own prospection of areas with potentially suitable habitats. The Morales grasshopper is primarily distributed in the south side of the Pyrenees (Spanish Pyrenees) and a small area located in the northeastern part of these mountains (French Pyrenees). The latter portion of the species distribution was intensively prospected during our field work but we only found a single population in the area (Err, France; Table 1). Overall, we were able to collect individuals from populations located across almost the entire climatic and elevation gradient occupied by the species (Table 1; Additional file 1: Figure S1), including populations from a wide range of habitats (from submediterranean shrub formations to subalpine grasslands) and differing in up to ~ 900 m of elevation (Table 1). We preserved specimens in 2 ml vials with 96 % ethanol and stored at − 20 ° C until DNA extraction. Population codes and more information on sampling sites are in Table 1. Specimens were collected in public lands under license from ‘Gobierno de Aragón’, ‘Generalitat de Catalunya’ and ‘Ordesa y Monte Perdido National Park’.

**Table 1:** Geographical location, elevation and number of genotyped individuals (*n*) for the studied populations of the Pyrenean Morales grasshopper

Locality	Code	Latitude	Longitude	Elevation (m)	*n*
Torla	TOR	42.63860	−0.08196	1822	20
Nerín	NER	42.59047	0.01062	1623	20
Saravillo	SAR	42.56089	0.23046	1313	20
Chía	CHI	42.56833	0.41708	1900	20
Aspes	ASP	42.44307	0.58389	1361	20
Boi	BOI	42.47945	0.86732	2046	12
Perves	PER	42.35250	0.83709	1370	20
Carmeniu	CAR	42.37243	1.33766	1140	20
Err	ERR	42.42762	2.05956	1800	19
Creueta	CRE	42.30131	1.99350	1928	11
Rasos	RAS	42.14165	1.76461	1840	20

### Microsatellite genotyping and basic genetic statistics

We extracted genomic DNA from a hind-leg of each individual using a salt extraction protocol [40]. We amplified and genotyped each individual using the 18 microsatellites markers described in [41]. We performed PCR amplifications following the procedure described in [14], run PCR products on an ABI 310 Genetic Analyzer (Applied Biosystems, Foster City, CA, USA) and scored genotypes using GENEMAPPER 3.7 (Applied Biosystems, Foster City, CA, USA).

We tested for deviations from Hardy-Weinberg Equilibrium (HWE) using exact tests [42] based on 900 000 Markov chain iterations and 100 000 dememorization steps as implemented in the program ARLEQUIN 3.5 [43]. We discarded two loci from all downstream analyses because of heterozygosity departure from HWE in all populations, probably due to the presence of null alleles according to MICRO-CHECKER analyses [44]. We also used Arlequin to test for linkage disequilibrium using a likelihood-ratio statistic, whose distribution was generated with 10 000 permutations. We did not find evidence for linkage disequilibrium between any pair of loci in any sampling population after sequential Bonferroni corrections [45].

### Genetic structure analyses

We estimated population genetic differentiation calculating *F*_ST_-values between all pairs of sampling populations and testing their significance with Fisher’s exact test after 10 000 permutations using ARLEQUIN 3.5. We corrected *P*-values using a sequential Bonferroni adjustment [45]. Due to the high frequency of null alleles in Orthoptera (e.g.*,* [14, 46]), we also calculated pairwise *F*_ST_-values corrected for null alleles (*F*_ST_NA) using the so-called ENA-method implemented in the program FREENA [47].

We inferred genetic structure using Bayesian clustering analyses in STRUCTURE 2.3.3 [48, 49]. We performed an iterative approach to assess the hierarchical genetic structure that could underlie broad genetic clustering patterns identified by STRUCTURE analyses including all populations (for a similar approach, see [50]). After an initial global analysis including all populations, we analyzed subsequent subsets of the data corresponding to the respective genetic clusters identified in the previous hierarchical level. For all analyses, we considered correlated allele frequencies and an admixture model without prior information on population origin. We performed ten independent runs for each value of *K* (1 to 12 for the complete dataset; and 1 to *n* + *x* for reduced datasets of *n* populations, being *x* a number to achieve at least three Δ*K* values) with a burn-in period of 200 000 steps and a run length of 1 000 000 Markov chain Monte Carlo (MCMC) cycles. We estimated the best-supported number of genetic clusters with the log probability of the data [Ln Pr (X|*K*)] [48] and the Δ*K* method [51]. We used the ‘*full search*’ algorithm in the program CLUMPP 1.1.2 [52] to align replicated runs and average individual assignment probabilities for the most likely *K-*value. Finally, we used DISTRUCT 1.1 [53] to produce bar plots displaying probabilities of individual membership to each inferred genetic cluster.

Complementary to the Bayesian clustering method, we used a discriminant analysis of principal components (DAPC) to identify clusters of genetically related individuals [54]. Although both clustering methods exhibit similarities (e.g. they do not require *a priori* delimitation of populations), several important differences exist in their analytical approaches. STRUCTURE suffers from the assumption of HWE and gametic disequilibrium [49] and typically fails to detect isolation-by-distance (IBD) [54] and hierarchical patterns of spatial genetic structure [51]. However, the multivariate analyses implemented in DAPC do not lay on the assumptions of STRUCTURE Bayesian analyses and could be more efficient to detect complex patterns of genetic differentiation [54]. DAPC is a methodological approach that requires data transformation using a principal component analysis (PCA) as a prior step to a discriminant analysis (DA). DA partitions genetic variation maximizing differences between clusters while minimizing within-cluster variation. We implemented DAPC analysis in R 3.1.2 using the package ADEGENET [54, 55]. At first, we used the ‘*find.cluster*’ function using all available principal components (PCs) to determine the best-supported number of genetic clusters using the Bayesian Information Criterion (BIC). The ‘*find cluster*’ function runs successive *K*-means clustering with increasing number of clusters (*K*) and provides a BIC value for each simulated *K*-value (i.e. *K*-value with the lowest BIC is the ‘optimal’ number of clusters). Secondarily, we determined the optimal number of PCs for the DAPC by cross-validation using the ‘*xvalDapc’* function with 100 replicates. We selected the number of PCs associated with the lowest ‘root mean squared error’ (RMSE) value. We ran DAPC using all the available discriminant functions and calculated the assignment probability of individuals to each cluster, which were represented with barplots using DISTRUCT.

We constructed a phylogenetic tree to evaluate the genetic relationships among all populations. We used the program POPULATIONS 1.2.31 [56] to obtain a neighbor-joining (NJ) tree based on pairwise Cavalli-Sforza and Edwards (*D*_*c*_) genetic distances [57]. This genetic distance is the most accurate to yield the correct tree topology for microsatellite markers under a variety of evolutionary scenarios without making assumptions in relation to mutation rates or constant population sizes [58].

### Climate niche modelling

We used climate niche models (CNMs) at different time periods to investigate whether current and past climate suitability are relevant factors shaping observed patterns of genetic differentiation among populations of the Pyrenean Morales grasshopper. For this purpose, we built a CNM using the ‘maximum entropy presence-only’ algorithm implemented in MAXENT 3.3.3 [59, 60] based on current climate layers and using 50 cross-validation replicate model runs. We used a total of 47 occurrence points obtained from the literature [30, 31] and our own sampling. To construct the models, we used the 19 present-day bioclimatic variables available in WorldClim [61] and downloaded at 30 arc-sec (*c*. 1 km) resolution [62]. To obtain the distribution of the Pyrenean Morales grasshopper in the Last Glacial Maximum (LGM, *c*. 21 kya BP) and the Last Interglacial (LIG, *c*. 120–140 kya BP), we projected contemporary species-climate relationships to these periods. The LGM layers were based on the Community Climate System Model (CCSM3, [63]) from the Palaeoclimate Modelling Intercomparison Project Phase II (PMIP2, [64]). We downloaded LGM layers from WorldClim at 2.5 arc-min and interpolated to 30 arc-sec resolutions. LIG layers were based on [65] and downloaded from WorldClim at 30 arc-sec resolution. According to the suggestions from [66], we limited the geographic extent of the climate layers to an area approximately 20 % larger than the known distribution range of the species in order to avoid model over-fitting. We used multivariate environmental similarity surfaces (MESS) calculation to address the problems derived from projecting the current distribution into novel climates (i.e. LGM and LIG periods) [67]. We used this approach to identify and discard climate layers with areas where the predictions should be treated with caution, due to the variables are outside the range present in the training data (for more details see [68]). We carried out MESS analyses iteratively excluding one variable in each step until discarding all out of range LGM and LIG variables compared to present-day variables. Finally, we checked the reliability of our past and current climate models following two approaches. At first, we developed a new current climate model using the variables with greater 5 % importance (as selected by Jackknife of regularized training gain procedure) and we compared its similarity with the current model generated by MESS analyses. Second, we developed a new model including only the most informative variable (Bio 1) and we compared its LGM and LIG projections with those obtained by MESS analyses (for a similar approach, see [69, 70]). All the output maps from the models were visualized using threshold values based on maximum training sensitivity plus specificity (MTSS).

### Topographic complexity

In order to investigate the role of topographic complexity (TC) as a potential factor shaping patterns of genetic differentiation, we calculated the surface ratio index for each cell from a digital elevation model using ‘DEM SURFACE TOOLS’ [71] in ARCGIS 10.0 (ESRI, Redlands, CA, USA). Surface ratio is an index of topographic complexity, with values close to one indicating flat areas and values higher than one indicating an abrupt relief and deep slopes [72]. We made calculations on a 90 m resolution digital elevation model from NASA Shuttle Radar Topographic Mission (SRTM Digital Elevation Data, [73]) and the final layer was transformed to 30 arc-sec (*c*. 1 km) resolution for subsequent analyses. Additionally, we used the digital elevation model to calculate a matrix of differences in elevation between each pair of studied populations (i.e. an elevation dissimilarity matrix).

### Environmental characterization of populations

In order to analyze the potential role of environment as a driving factor of genetic differentiation, we characterized the environmental space of each population using a principal component analysis (PCA) with ‘varimax’ rotation applied to the values of the 19 present-day bioclimatic variables from WorldClim extracted from sampling sites, occurrence points used in MAXENT and 1 000 randomly distributed points in the study area. This procedure allowed us to capture the environmental variation of the study area and avoid potential bias resulting from just considering environmental conditions from the sampling sites. Then, we obtained for each population the PC scores of the first three PCs, which explained the 73.18, 10.92 and 8.38 % respectively of the environmental variance (Additional file 1: Figure S1). Finally, we calculated environmental dissimilarity between each pair of populations using Euclidean distances for the obtained PC scores using the ‘*dist*’ function in R. We performed PCA analysis using IBM SPSS 21.0 (IBM Coorp., Armonk, NY, USA).

### Isolation by resistance matrices

We applied circuit theory to model gene flow between populations and test the effects of different landscape resistance scenarios (IBR) on observed patterns of genetic differentiation [12, 74]. We used CIRCUITSCAPE 4.0 [11] to calculate resistance distance matrices between all pairs of populations considering an eight-neighbor cell connection scheme. We obtained different IBR distance matrices considering as inputs in CIRCUITSCAPE the following raster layers: current, LGM and LIG climate niche suitability and topographic complexity. We assigned pixel values of climate niche suitability maps as conductance values and pixel values of topographic complexity layers as resistance values. We also used CIRCUITSCAPE to test for the effect of isolation-by-distance (IBD) by calculating pairwise resistance distances on a completely ‘flat’ landscape based on a raster layer in which all cells had an equal value (conductance = 1). This IBD resistance model yields similar results than a matrix of Euclidean geographical distances, but it is more appropriate for comparison with others competing models also generated with CIRCUITSCAPE [75,^76^].

### Multiple matrix regression with randomization

All IBR matrices were tested against genetic distance matrices (pairwise *F*_ST_ and *F*_ST_NA-values) using multiple matrix regressions with randomization (MMRR, [24]) as implemented in R. In these models, we also included elevation (ELEV_DIS_, see ‘Topographic complexity’ section) and climate dissimilarity (CLIM_DIS_, see ‘Environmental characterization of populations’ section) distance matrices in order to test a possible pattern of IBE. We used a backward procedure to select final models, removing non-significant variables from an initial full model including all explanatory predictors. We tested the significance of the remaining variables again until no additional term reached significance (e.g.*,* [77]).

### Distance-based redundancy analysis

Complementary to MMRR analyses, we tested the relationship between genetic differentiation, geography and environment using distance-based redundancy analyses (dbRDA, [38]). This approach is based on a multivariate multiple regression and estimates the percentage of genetic variation explained by a given predictor or set of predictors. We performed dbRDA using the ‘*capscale*’ function in the package VEGAN [78] as implemented in R. The genetic distance matrix (pairwise *F*_ST_ or *F*_ST_NA-values) was tested against the following variables: i) geographic distances (IBD), ii) elevation and iii) population’s PC scores of the first three axes from the PCA performed on the environmental data (see ‘Environmental characterization of populations’ section). Euclidean geographical distances between sampled populations were calculated using GEOGRAPHIC DISTANCE MATRIX GENERATOR 1.2.3 [79]. Geographic distances were tested after transforming the Euclidean geographical distance matrix to a continuous rectangular vector by principal coordinates analyses (PCoA) using the ‘*pcnm*’ function in the package VEGAN. Significance of the predictors was assessed using multivariate *F*-statistics with 9999 permutations using the ‘*anova.cca*’ function included in the package VEGAN. We first analyzed the relationship between the genetic distance matrix and each variable separately (marginal test) and then we performed a partial dbRDA (conditional test) for each variable while controlling for the influence of geography (fitted as covariate).

### Geostatistical simulations and Bayesian inference

We quantified the relative effects of geography and environment on genetic differentiation using SUNDER [25], a novel geostatistical method modelling covariance in allele frequencies between populations as a decreasing function of geographical and ecological distances ([25], see also [80]). SUNDER uses a Bayesian framework with a MCMC algorithm to estimate the magnitude of the effects of these variables and implements a model selection procedure by cross-validation to assess which sub-model (e.g. with or without the effect of environment) best fits to the data. Using the multinomial model, we ran SUNDER with 10 million of iterations for each data set, sampling every 1 000 iterations. We set to update in the MCMC iterations all parameters of the vector θ (α, variance of allele frequencies; *β*_*G*_ and *β*_*E*_, magnitude of the effect of geography and environment respectively on genetic covariance; γ, smoothness of spatial variation of allele frequencies; δ*,* variation in the allele frequency of a population departing from the other populations). We also set their initial state and upper bounds of their Dirichlet prior distributions following suggestions in [25]. We visually checked trace plots for parameters to assure convergence. We used the 10 % of our data set (sites × locus) as validation set during the cross-validation procedure. We performed SUNDER analyses using as environmental matrices the elevation dissimilarity matrix (ELEV_DIS_) and the climate dissimilarity distance matrix (CLIM_DIS_), which we separately tested against IBR distance matrix based on a completely ‘flat’ landscape (IBD). Before analyses, we standardized all distance matrices by their respective standard deviations.

## Results

### Climate niche modelling

**Fig. 3:**
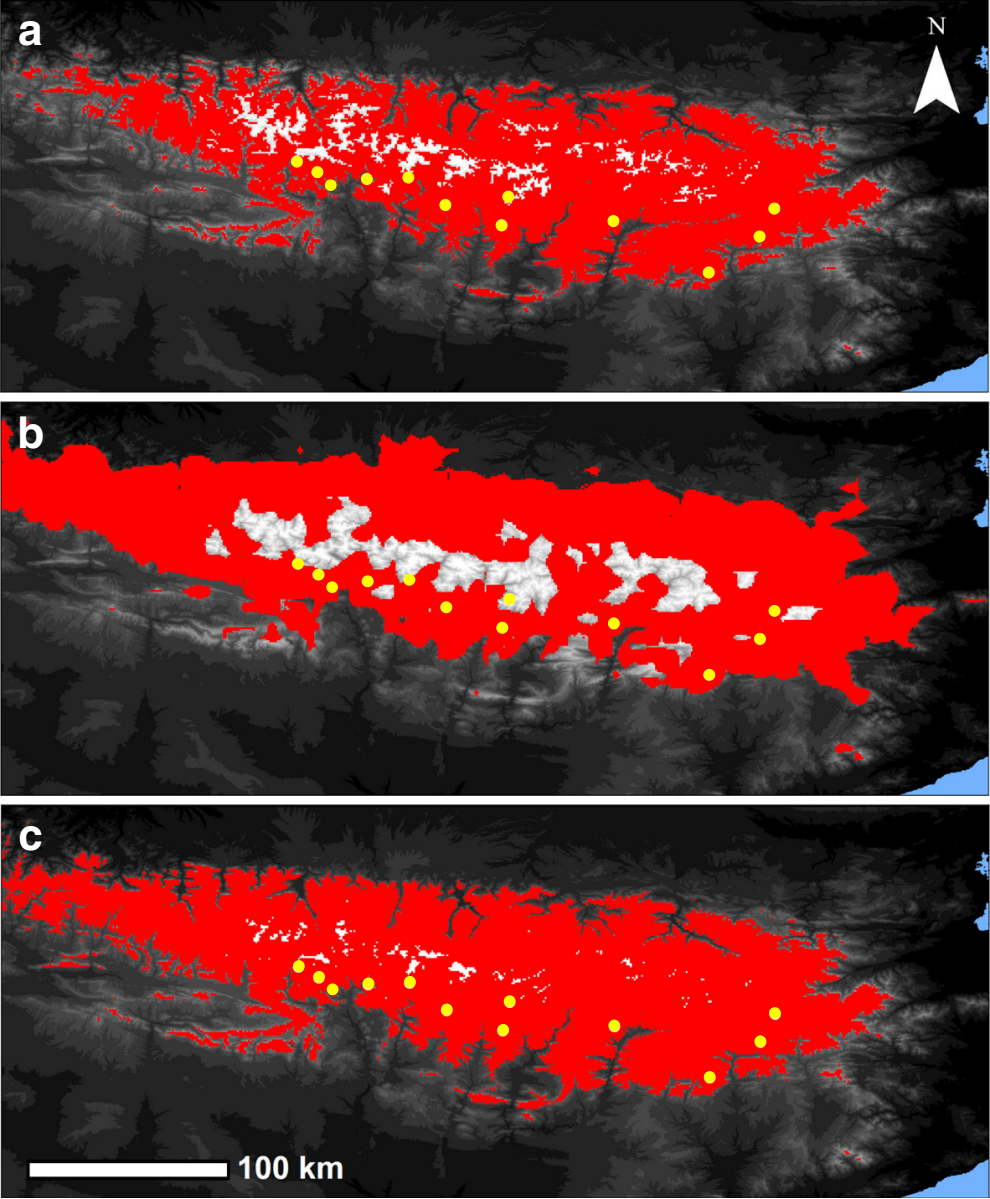
Climate niche modelling of the Pyrenean Morales grasshopper for (**a**) the present, (**b**) the Last Glacial Maximum (LGM, *c*. 21 kya BP) and (**c**) the Last Interglacial (LIG; *c*. 120–140 kya BP). Climatically suitable areas defined using the maximum training sensitivity plus specificity threshold (MTSS) is in red. The topography is in the background map, in which whiter colors indicate higher elevations and darker colors indicate lower elevations (range from 0 to 3404 m.a.s.l.). Yellow dots represent the eleven sampling sites

We constructed past and present-day final climate niche models using six bioclimatic variables: annual mean temperature (Bio1), mean temperature of the coldest quarter (Bio11), annual precipitation (Bio12), precipitation of the driest month (Bio14), precipitation seasonality (Bio15), and precipitation of the warmest quarter (Bio18). This model had a very high value of area under the receiving operator characteristics curve (AUC = 0.919 ± 0.067 SD), indicating overall good performance. The predicted habitat suitability area in the present was consistent with the current distribution of the species, but some areas in the northern and west side of the Pyrenees where the Morales grasshopper has not been recorded were also predicted to be suitable for the species (Fig. 3a). Projections of the present-day climate niche envelope to the LGM and LIG suggesting that the Pyrenean Morales grasshopper has experienced important distributional shifts during the Pleistocene. During the LGM, areas above 1 800–2 000 m.a.s.l. resulted unsuitable for the species and its potential distribution range expanded to areas of lower altitude across the western and northern side of the Pyrenees (Fig. 3b). Conversely, the potential distribution range of the species during the LIG expanded to higher altitudes but its peripheral geographical limits were similar than in the present (Fig. 3c).

### Population genetic structure

Pairwise *F*_ST_-values ranged from 0.021 to 0.216 and 53 of 55 comparisons were significant after sequential Bonferroni correction (Additional file 1: Table S1). Pairwise *F*_ST_NA-values were lower than *F*_ST_-values and ranged from 0.014 to 0.148. Pairwise *F*_ST_-values were highly correlated with pairwise *F*_ST_NA-values (Mantel *r* = 0.941; *P* < 0.001).

STRUCTURE analyses on all populations showed that the best-supported number of clusters was *K* = 2 according to the Δ*K* method (Fig. 2b; Additional file 1: Figure S2). These initial analyses detected a strong correspondence between the inferred genetic clusters and their geographic location, even when a broader range of *K*-values (*K* = 2–7) was evaluated. Subsequent hierarchical analyses on different subsets of populations detected further genetic structuring (Fig. 2b). Individual assignment probabilities to a certain genetic cluster were generally high and the distribution of the hierarchical genetic structure exhibited congruence with the geographical location of the studied populations. Consecutive hierarchical analyses revealed that each population constituted a single cluster, although many pairs of populations showed a considerable degree of genetic admixture (Fig. 2b).

**Fig. 4:**
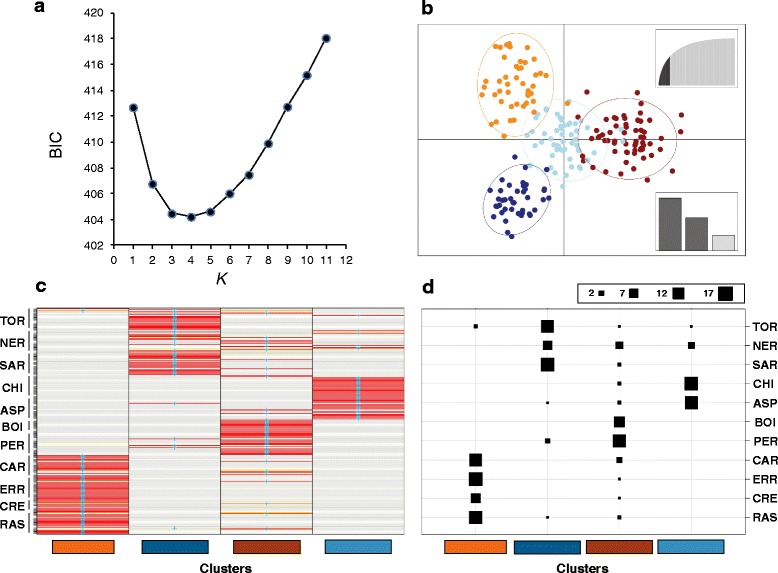
Summary of the results of discriminant analysis of principal components (DAPC). Panel **a** represents the Bayesian Information Criterion (BIC) for each value of *K*. The minimum value of BIC before the first increase or stabilization indicates the best-supported number of genetic clusters (*K* = 4 in this case). Panel **b** represents an ordination plot for the first two discriminant axes. Each dot represents one individual and colors and inertia ellipses indicate their assignment to one of the four genetic clusters inferred by DAPC. The up-right graph inset displays the variance explained by the principal component axes used for DAPC (*in dark grey*). The bottom-right inset displays in relative magnitude the variance explained by the two discriminant axes plotted (*in dark grey*). Panel **c** represents whether the individuals (*rows*) were correctly assigned (based on discriminant functions) to the genetic cluster where they were included a priori (*columns*) by *K*-means analyses used to infer the best-supported clustering solution. Colors represent membership probabilities to each genetic cluster (*red* = 1, *orange* = 0.75, *yellow* = 0.25, *white* = 0) and blue crosses indicate the cluster where the individuals were originally assigned by *K*-means analyses. Generally, the DAPC classification of individuals is consistent with their assignment to the clusters originally identified by *K*-means analyses (i.e. *blue crosses are on red rectangles*). Panel **d** shows the number of individuals from each population (*rows*) assigned to each of the four inferred genetic clusters (*columns*). The size of black squares is proportional to the number of individuals assigned to the different clusters (*up-right legend*). Population codes are described in Table 1

**Fig. 5:**
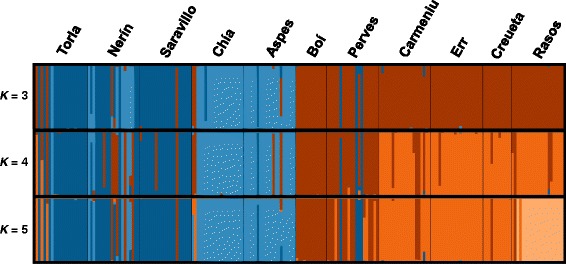
Results of clustering analyses for 202 individuals of the Pyrenean Morales grasshopper for different numbers of genetic clusters (*K*) based on a discriminant analysis of principal components (DAPC). Each individual corresponds to a vertical bar partitioned into *K*-colored segments that represent the individual’s probability of belonging to the cluster with that color. Black lines separate individuals from different populations. Cluster colors in *K* = 4 barplot correspond to those of Fig. 4

DAPC analyses identified also a deep spatial genetic structure in concordance with the geographic location of populations. The minimum BIC values were obtained for *K* = 3–5 (Fig. 4a). Considering *K* = 4 (the *K*-value with the lowest BIC value), DAPC partitioned all individuals in western (TOR-NER-SAR populations), central-west (CHI-ASP), central-east (BOI-PER) and eastern (CAR-ERR-CRE and RAS) groups (Fig. 5). Discriminant functions based on DAPC analyses correctly assigned most individuals to the genetic cluster where they were assigned a priori by *K*-means analyses used to infer the best-supported clustering solution [54] (Fig. 4c). The low overlapping of the genetic clusters on the ordination plot indicated high degree of differentiation between them (Fig. 4b). When *K* = 3 and *K* = 5 were considered and compared with *K* = 4, DAPC revealed a hierarchical distribution of genetic variation similar to that identified by STRUCTURE (Fig. 2b and Fig. 5).

The result of the NJ tree based on *D*_*c*_ genetic distances showed that populations were included into monophyletic groups geographically clustered according to the hierarchical structure inferred by STRUCTURE and DAPC analyses (Fig. 2a, b and Fig. 5).

### Multiple matrix regression with randomization

**Fig. 6:**
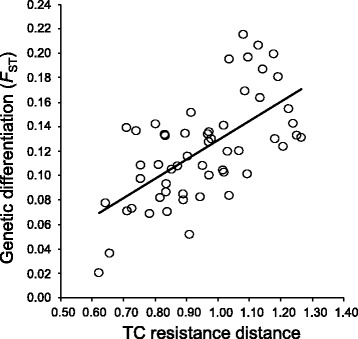
Relationship between genetic differentiation (*F*_ST_) and topographic complexity (TC) resistance distances (IBR_TC_) (calculated using CIRCUITSCAPE) in eleven populations of Pyrenean Morales grasshopper. Regression line is shown

Genetic differentiation (*F*_ST_) was positively associated with geographic distance (i.e. IBD, resistance distances based on a completely ‘flat’ landscape), topographic complexity (IBR_TC_) and current (IBR_CURRENT_), LGM (IBR_LGM_) and LIG (IBR_LIG_) habitat resistance distances when these variables were included alone into different models (all *P*s < 0.012). Indeed, Mantel tests showed that all these variables were highly inter-correlated (Additional file 1: Table S2). Climatic dissimilarity (CLIM_DIS_) was also correlated with all other variables, but with comparatively lower Mantel *r* values, whereas elevation dissimilarity (ELEV_DIS_) was only significantly correlated with CLIM_DIS_ (Additional file 1: Table S2). Univariate models including IBD and IBR_TC_ provided the highest and most similar coefficients of determination (*r*^2^) (Table 2). However, only IBR_TC_ was included into the final model after the backward selection procedure (Fig. 6). The rest of variables did not remain significant when they were tested against topographic complexity (all *P*s > 0.13). Analyses based on *F*_ST_NA yielded similar results, but models had generally lower values of coefficient of determination (*r*^2^) (Table 2). Our results remained similar after sequential Bonferroni correction for multiple testing [45].

**Table 2:** Results of univariate matrix regressions with randomization for genetic differentiation (*F*_ST_ and *F*_ST_NA-values corrected for null alleles) among eleven populations of the Pyrenean Morales grasshopper in relation with elevation (ELEV_DIS_) and climatic (CLIM_DIS_) dissimilarity and five isolation by resistance (IBR) scenarios: IBD, isolation by distance (i.e. equal resistance to all pixel values, equivalent to geographical distance); IBR_TC_, topographic complexity; IBR_CURRENT_, current habitat suitability; IBR_LGM_, Last Glacial Maximum habitat suitability and IBR_LIG_, Last Interglacial habitat suitability

	*F*_ST_				*F*_ST_ NA			
	*r* ^2^	β	*t*	*P*	*r* ^2^	β	*t*	*P*
**IBD**	0.390	0.92	5.82	0.001	0.332	0.83	5.13	0.001
**IBR**_**TC**_	0.391	0.92	5.83	0.001	0.335	0.83	5.16	0.001
**IBR**_**CURRENT**_	0.235	0.47	4.03	0.005	0.198	0.41	3.61	0.006
**IBR**_**LGM**_	0.162	0.42	3.20	0.012	0.259	0.52	4.30	0.002
**IBR**_**LIG**_	0.308	0.56	4.85	0.001	0.251	0.49	4.21	0.002
ELEV_DIS_	0.008	−0.07	−0.64	0.509	0.013	−0.09	−0.83	0.397
CLIM_DIS_	0.069	0.25	1.98	0.075	0.041	0.18	1.51	0.132

Predictors with *P* < 0.05 in bold

### Distance-based redundancy analysis

Marginal tests showed a significant association between genetic differentiation (*F*_ST_ and *F*_ST_NA-values) and geography and one environmental predictor (climate PC2) that explained 46.80–53.66 % and 40.12–45.70 % of genetic variation, respectively (Table 3). Climate PC2 was explained by a pool of bioclimatic variables related to annual temperature variation (Bio2, Bio3 and Bio7; Additional file 1: Table S3). However, the environmental predictor (climate PC2) remained not significant after accounting for the influence of geography in the conditional test (Table 3). Our results remained similar after sequential Bonferroni correction [45]. Analyses based on *F*_ST_NA-values (Table 3) or performed with PCA considering only the six bioclimatic layers employed to build the climate niche model in MAXENT provided similar results (data not shown).

**Table 3:** Results of distance-based redundancy analyses (dbRDA) testing the effects of geography, climate and elevation on genetic differentiation among eleven populations of the Pyrenean Morales grasshopper

Marginal tests				Conditional tests			
Variable	*F*	*P*	% var	Variable	*F*	*P*	% var
*F*_ST_							
**Geography**	10.42	0.001	53.66				
Elevation	0.54	0.651	5.75	Elevation	0.48	0.844	2.64
Climate PC1	0.67	0.581	7.00	Climate PC1	0.42	0.883	2.35
**Climate PC2**	7.57	0.002	45.70	Climate PC2	0.62	0.720	3.35
Climate PC3	1.59	0.194	15.06	Climate PC3	2.02	0.084	9.37
*F*_ST_ NA							
**Geography**	7.91	0.001	46.80				
Elevation	0.48	0.747	5.14	Elevation	0.43	0.871	2.76
Climate PC1	0.61	0.661	6.40	Climate PC1	0.27	0.961	1.79
**Climate PC2**	6.02	0.005	40.12	Climate PC2	0.70	0.665	4.33
Climate PC3	1.84	0.144	17.05	Climate PC3	2.13	0.073	11.22

Geography is tested after transforming the Euclidean geographical distance matrix to a continuous rectangular vector by principal coordinates analyses (PCoA). In marginal tests, we tested each predictor separately, while in conditional (partial) tests geography was always included as covariate. The proportion of the multivariate genetic variation explained (% var) by a given predictor or set of predictors is indicated. Predictors with *P* < 0.05 in bold

### Geostatistical simulations and Bayesian inference

SUNDER analyses indicated that the models that best fit to the genetic data were those exclusively including the geographic component (Table 4). The Bayesian posterior estimates of the parameter *β*_*G*_ (representing the magnitude of the effect of geography) were smaller than *β*_*E*_ (representing the magnitude of the effect of environment), indicating a primordial effect of geographical distances and an absence of isolation by climate or elevation on genetic differentiation (Table 4).

**Table 4:** Results of Bayesian inference and model selection in SUNDER testing the relative effects of geographical and environmental variables on genetic differentiation among eleven populations of the Pyrenean Morales grasshopper

		Likelihood and (*β*_*i*_) for each model		
		G	E	G + E
Environmental variable	CLIM_DIS_	**−469.43** **(** ***β***_***G***_ **= 14.95)**	−471.44 (*β*_*E*_ = 25.44)	−469.78 (*β*_*G*_ = 25.50) *(β*_*E*_ = 21.31)
	ELEV_DIS_	**−460.11** **(** ***β***_***G***_ **= 23.01)**	−461.24 (*β*_*E*_ = 31.08)	−460.63 (*β*_*G*_ = 17.45) *(β*_*E*_ = 31.11)

We separately tested the environmental variables [elevation (ELEV_DIS_) and climatic (CLIM_DIS_) dissimilarity matrices] against an IBD resistance distance matrix (i.e. equal conductance to all pixel values, equivalent to geographic distance). ‘G’ corresponds to models only considering geography, ‘E’ corresponds to models only considering the environmental variable, and ‘G + E’ corresponds to models considering both of them. For all runs, we show the likelihood of each model based on the validation dataset and the values of *β*_*i*_ parameter. The *β*_*i*_ parameter quantifies the magnitude of the effect of each variable on genetic covariance (small values correspond to a strong decreasing of the genetic covariance with increasing geographical or environmental distance, i.e. small values indicate an important effect of such variable). The most likely model for each comparison is in bold

## Discussion

Despite its small distribution range (<200 km between the most distant populations), the Pyrenean Morales grasshopper exhibits a strong genetic structure congruent with the geographical location of its different populations. Our microsatellite-based clustering and phylogenetic analyses revealed a strong hierarchical structure and a relatively low degree of inter-group genetic admixture. In most cases, the distinct genetic clusters corresponded to single populations, which clustered in the hierarchically superior genetic group according to the main mountain chains of the region. In comparison with other Mediterranean orthoptera, the Pyrenean Morales grasshopper has a population genetic structure as remarkable as that found at a wider spatial scale in *Mioscirtus wagneri* (global *F*_ST_ ~ 0.19) and much higher than that shown for *Ramburiella hispanica* at a similar spatial scale (global *F*_ST_ ~ 0.02), two species presenting a patchy distribution restricted to highly isolated and fragmented habitats [14, 81, 82]. The genetic structure found in the Pyrenean Morales grasshopper is in accordance with isolation driven by geographical factors, in which the presence of deep barriers disrupting gene flow between populations primarily explained levels of genetic differentiation [69]. Despite climate warming during LIG and present has probably led to upward altitudinal shifts in the species distribution, its populations have presented a continuous distribution and exhibited a similar connectivity during both cold and warm periods characterizing the last 120 kya. Our niche models suggest that only a few contemporary populations probably went extinct during the LGM, but this might have had a little impact on global patterns of genetic structure if nearby populations, with a similar genetic makeup, colonized present-day suitable habitat patches. The absence of the species in areas identified as climatically suitable and stable during the last 120 kya according to our niche models (i.e. large areas in the peripheral northern and western portion of the Pyrenees) could be linked to historical events (such as extinctions) predating the temporal scale addressed in our study [83]. It could also depend on constraints of our climate models not capturing other important abiotic or biotic interactions that may contribute to the distribution of the species in those areas [84]. Thus, our analyses suggest that the strong genetic structure found across the species distribution range has not arisen as consequence of long-term isolation driven by Pleistocene climatic oscillations that shaped range-wide patterns of genetic structure in many other taxa from temperate regions [34, 85, 86].

Our different landscape genetic approaches confirmed that neutral divergence resulted from the isolating effects of topography primarily drove the deep patterns of population genetic differentiation observed in the Morales grasshopper. The resistance model based on topographic complexity was the best fit to our data, indicating that physical features defining the abrupt landscape characterizing the Pyrenees shaped genetic differentiation. In particular, deep valleys with a north-south orientation and slopes that are generally steep and create canyons and ridges on the landscape crisscross the central and eastern portion of the Pyrenees inhabited by Morales grasshopper. Hence, these topographic features could become impassable barriers and restrict gene flow as has been previously documented for other species with limited dispersal capacities and inhabiting regions of remarkable topographical roughness [35, 36, 87, 88]. The remaining analyzed landscape factors, such as resistance-based distances informed by current and past climate niche models, did not show either a significant association with genetic differentiation after controlling for the influence of topographic complexity or geographical distances. The lower explanatory power of CNM-based resistance distances may be related with the fact that they are not capturing well microhabitat structure, which has been found to be highly relevant in determining the distribution and demography of grasshoppers [89–92]. The short generation time of the studied species (=1 year) may have also resulted in contemporary patterns of genetic differentiation are not capturing the genetic signal left by dispersal routes defined by habitat corridors during the past. This fact contrasts with patterns found in species with longer generation times and that are likely to show a time lag in their response to changing climatic conditions [77, 93].

We found no support for ecological divergence and local adaptation processes have contributed to population genetic differentiation and the three different employed approaches (MMRR analysis using dissimilarity matrices, dbRDA analysis using raw variables and Bayesian inference) confirmed the consistency of this result. We did not find support either for altitude as an isolating mechanism despite elevation gradients have been previously found to be a significant driver of genetic and phenotypic variation in grasshoppers and many other organisms [94, 95]. Our results contrasts with other studies that have documented an important role of environment on genetic differentiation in many taxa [4, 18], including some insects such as grasshoppers [96, 97], walking sticks [98] or beetles [99]. This discrepancy may be in part due to these studies considered species exhibiting narrow feeding preferences and analyzed ecological dissimilarity in terms of host-plant associations, an aspect that may have a higher impact on genetic divergence than the climate or elevation gradients considered in our study [96–99]. The meta-analysis by [18] showed that isolation-by-ecology is more frequent than IBD in insects, particularly in species with strong patterns of genetic structure. Considering the high degree of genetic differentiation among our study populations, we can discard the hypothesis that a high level of gene flow has counteracted the potential disruption of gene flow driven by local adaptation processes mediated by environmental heterogeneity [17]. Hence, the lack of effects of environment on gene flow could be due to different reasons, including low environmental variation among sampling sites [5] or local adaptation driven by other ecological factors not considered in this study (e.g. distinct host plants or habitat structure [96]).

## Conclusions

This study emphasizes the importance of examining jointly different scenarios of population isolation to understand their contribution to the spatial distribution of genetic variation across a species range. Our analyses evidence the importance of topographic complexity in determining patterns of genetic differentiation, indicating that limited dispersal and drift, due to scarce population connectivity, is shaping the genetic structure found in our study system (e.g., [86]). Further research harnessing high-throughput sequencing will provide a better understanding about the potential association between loci under selection and different ecological factors, which may help to identify genomic regions involved in local adaptation processes [15, 100]. Exploring the relationship between environmental features and genetic and phenotypic patterns of variation could also provide insights about the potential interplay of evolutionary and ecological processes in shaping range-wide patterns of genetic differentiation [101, 102].

## Ethics

This study did not require ethical approval. Specimens were collected under license from ‘Gobierno de Aragón’, ‘Generalitat de Catalunya’ and ‘Ordesa y Monte Perdido National Park’. Our sampling procedures did not affect the survival of the studied populations.

## Availability of data and materials

Nuclear microsatellite data are available in the LabArchives repository (http://dx.doi.org/10.6070/H4QC01JB). All other data supporting the results of this article are included within the article and its additional files.

## Consent to publish

Not applicable.

## Additional file

Additional file 1: Table S1. Genetic differentiation between eleven populations of the Pyrenean Morales grasshopper (*Chorthippus saulcyi moralesi*). **Table S2.** Results of Mantel tests analyzing the relationship between the different distance matrices (predictors) used to evaluate the factors associated with population genetic differentiation in the Pyrenean Morales grasshopper (*Chorthippus saulcyi moralesi*). **Table S3.** Results of principal component analysis (PCA) applied to the values of the 19 present day bioclimatic variables obtained from the WorldClim dataset. **Figure S1.** The position in the environmental space (first two principal components of a PCA based on the 19 bioclimatic variables from the WorldClim dataset) of all known populations of the Pyrenean Morales grasshopper (*Chorthippus saulcyi moralesi*). **Figure S2.** Results of Bayesian clustering analyses in STRUCTURE to determine the best-supported number of clusters in hierarchical analyses. (DOCX 871 kb)

## Abbreviations

AUC: area under the curve.
BIC: Bayesian information criterion
BP: before present
CNM: climate niche models
DAPC: discriminant analysis of principal components
dbRDA: distance-based redundancy analyses
HWE: Hardy-Weinberg equilibrium
IBD: isolation-by-distance
IBE: isolation-by-environment
IBR: isolation-by-resistance
kya: thousand years ago
LGM: last glacial maximum
LIG: last interglacial
m.a.s.l.: meters above sea level
MCMC: Markov chain Monte Carlo
MMRR: multiple matrix regressions with randomization
MTSS: maximum training sensitivity plus specificity threshold
NJ: neighbor-joining
PCA: principal component analysis
PCoA: principal coordinates analyses
RMSE: root mean squared error
