# Supplementary material for: Hierarchical genetic structure shaped by topography in a narrow-endemic montane grasshopper
Source: BMC Evol Biol. 2016 May 5;16:96. doi: 10.1186/s12862-016-0663-7 (PMC4858822; doi:10.1186/s12862-016-0663-7)
Supplement: Additional file 1: Table S1. — Genetic differentiation between eleven populations of the Pyrenean Morales grasshopper (Chorthippus saulcyi moralesi). Table S2. Results of Mantel tests analyzing the relationship between the different distance matrices (predictors) used to evaluate the factors associated with population genetic differentiation in the Pyrenean Morales grasshopper (Chorthippus saulcyi moralesi). Table S3. Results of principal component analysis (PCA) applied to the values of the 19 present day bioclimatic variables obtained from the WorldClim dataset. Figure S1. The position in the environmental space (first two principal components of a PCA based on the 19 bioclimatic variables from the WorldClim dataset) of all known populations of the Pyrenean Morales grasshopper (Chorthippus saulcyi moralesi). Figure S2. Results of Bayesian clustering analyses in STRUCTURE to determine the best-supported number of clusters in hierarchical analyses. (DOCX 871 kb) [file 12862_2016_663_MOESM1_ESM.docx]

**ADDITIONAL FILE 1**

**BMC EVOLUTIONARY BIOLOGY**

**Title**

*Hierarchical genetic structure shaped by topography in a narrow-endemic montane grasshopper*

**Authors**:

Víctor Noguerales

Grupo de Investigación de la Biodiversidad Genética y Cultural,

Instituto de Investigación en Recursos Cinegéticos - IREC (CSIC, UCLM, JCCM),

Ronda de Toledo 12, E-13071 Ciudad Real, Spain.

E-mail: *[victor.noguerales@csic.es](mailto:victor.noguerales@csic.es)*

Pedro J. Cordero

Grupo de Investigación de la Biodiversidad Genética y Cultural,

Instituto de Investigación en Recursos Cinegéticos - IREC (CSIC, UCLM, JCCM),

Ronda de Toledo 12, E-13071 Ciudad Real, Spain.

E-mail: *[pedrojavier.cordero@uclm.es](mailto:pedrojavier.cordero@uclm.es)*

Joaquín Ortego

Department of Integrative Ecology,

Estación Biológica de Doñana, EBD-CSIC,

Avda. Américo Vespucio s/n, E-41092 Seville, Spain.

E-mail: *[joaquin.ortego@csic.es](mailto:joaquin.ortego@csic.es)*

**Table S1** Genetic differentiation between eleven populations of the Pyrenean Morales grasshopper (*Chorthippus saulcyi moralesi*). We present pairwise *F*_ST_ values below the diagonal and *F*_ST_NA values corrected for null alleles above the diagonal. FreeNA software used to calculate *F*_ST_NA values does not implement statistical tests of population genetic differentiation and these were only performed for *F*_ST_ values in Arlequin. *F*_ST_ values in bold are statistically significant after sequential Bonferroni correction. Population codes as in Table 1.

|  | TOR | NER | SAR | CHI | ASP | BOI | PER | CAR | ERR | CRE | RAS |
| --- | --- | --- | --- | --- | --- | --- | --- | --- | --- | --- | --- |
| TOR | - | 0.014 | 0.065 | 0.080 | 0.087 | 0.111 | 0.081 | 0.075 | 0.107 | 0.104 | 0.096 |
| NER | 0.021 | - | 0.057 | 0.059 | 0.076 | 0.095 | 0.066 | 0.076 | 0.103 | 0.103 | 0.098 |
| SAR | **0.070** | **0.074** | - | 0.104 | 0.114 | 0.127 | 0.083 | 0.105 | 0.136 | 0.139 | 0.127 |
| CHI | **0.106** | **0.083** | **0.140** | - | 0.043 | 0.114 | 0.105 | 0.094 | 0.136 | 0.141 | 0.120 |
| ASP | **0.116** | **0.108** | **0.143** | **0.072** | - | 0.105 | 0.110 | 0.103 | 0.148 | 0.141 | 0.134 |
| BOI | **0.131** | **0.109** | **0.135** | **0.133** | **0.109** | - | 0.055 | 0.085 | 0.109 | 0.093 | 0.112 |
| PER | **0.101** | **0.083** | **0.085** | **0.134** | **0.137** | **0.078** | - | 0.065 | 0.069 | 0.073 | 0.106 |
| CAR | **0.102** | **0.121** | **0.142** | **0.135** | **0.152** | **0.087** | **0.071** | - | 0.037 | 0.041 | 0.083 |
| ERR | **0.132** | **0.143** | **0.181** | **0.188** | **0.197** | **0.120** | **0.084** | **0.052** | - | 0.025 | 0.099 |
| CRE | **0.134** | **0.155** | **0.200** | **0.207** | **0.216** | **0.105** | **0.103** | **0.081** | 0.037 | - | 0.075 |
| RAS | **0.124** | **0.131** | **0.164** | **0.170** | **0.196** | **0.136** | **0.128** | **0.094** | **0.110** | **0.098** | - |

**Table S2** Results of Mantel tests analyzing the relationship between the different distance matrices (predictors) used to evaluate the factors associated with population genetic differentiation in the Pyrenean Morales grasshopper (*Chorthippus saulcyi moralesi*): elevation (ELEV_DIS_) and climatic (CLIM_DIS_) dissimilarity and five isolation by resistance (IBR) scenarios: IBD, isolation by distance (*i.e.* equal resistance to all pixel values, equivalent to geographical distance); IBR_TC_, topographic complexity; IBR_CURRENT_, current habitat suitability; IBR_LGM_, Last Glacial Maximum habitat suitability and IBR_LIG_, Last Interglacial habitat suitability. We present Mantel *r* values below the diagonal and *P*-values for each comparison above the diagonal. Significant *P*-values after sequential Bonferroni correction in bold.

|  | IBD | IBR_TC_ | IBR_CURRENT_ | IBR_LGM_ | IBR_LIG_ | ELEV_DIS_ | CLIM_DIS_ |
| --- | --- | --- | --- | --- | --- | --- | --- |
| IBD | - | **0.001** | **0.001** | **0.001** | **0.001** | 0.875 | **0.001** |
| IBR_TC_ | 0.990 | - | **0.001** | **0.001** | **0.001** | 0.877 | **0.001** |
| IBR_CURRENT_ | 0.924 | 0.930 | - | **0.001** | **0.001** | 0.946 | **0.001** |
| IBR_LGM_ | 0.620 | 0.625 | 0.629 | - | **0.002** | 0.648 | **0.003** |
| IBR_LIG_ | 0.976 | 0.977 | 0.962 | 0.584 | - | 0.882 | **0.001** |
| ELEV_DIS_ | -0.136 | -0.139 | -0.184 | -0.072 | -0.147 | - | **0.004** |
| CLIM_DIS_ | 0.641 | 0.642 | 0.691 | 0.474 | 0.669 | 0.479 | - |

**Table S3** Results of principal component analysis (PCA) applied to the values of the 19 present day bioclimatic variables obtained from the WorldClim dataset. We report factor loadings for the first three principal components (PC) and the 19 bioclimatic variables. Bold type indicates variables with factor loadings higher than 0.7.

|  | PC1 | PC2 | PC3 |
| --- | --- | --- | --- |
| Bio1 | **0.920** | 0.207 | 0.268 |
| Bio2 | 0.271 | **0.952** | 0.059 |
| Bio3 | -0.058 | **0.885** | 0.328 |
| Bio4 | 0.656 | 0.398 | -0.522 |
| Bio5 | **0.909** | 0.358 | 0.146 |
| Bio6 | **0.920** | 0.026 | 0.323 |
| Bio7 | 0.538 | **0.788** | -0.213 |
| Bio8 | **0.901** | 0.091 | -0.157 |
| Bio9 | **0.246** | 0.213 | **0.800** |
| Bio10 | **0.939** | 0.218 | 0.196 |
| Bio11 | **0.911** | 0.136 | 0.333 |
| Bio12 | **-0.899** | -0.412 | 0.066 |
| Bio13 | **-0.814** | -0.512 | 0.145 |
| Bio14 | **-0.922** | -0.336 | -0.109 |
| Bio15 | **0.874** | -0.020 | -0.005 |
| Bio16 | **-0.860** | -0.449 | 0.102 |
| Bio17 | **-0.924** | -0.349 | 0.013 |
| Bio18 | **-0.816** | -0.475 | -0.153 |
| Bio19 | **-0.898** | -0.232 | 0.318 |

**Fig. S1** The position in the environmental space (first two principal components of a PCA based on the 19 bioclimatic variables from the WorldClim dataset) of all known populations of the Pyrenean Morales grasshopper (*Chorthippus saulcyi moralesi*). Sampling sites used in genetic analyses, occurrence points used to build the Climate Niche Models (CNM) in Maxent and the 1000 random points used to perform the PCA are represented with red, blue and white open dots, respectively. Population codes as in Table 1.

**Fig. S2** Results of Bayesian clustering analyses in Structure to determine the best-supported number of clusters in hierarchical analyses including (i) all populations or (ii - viii) different subsets of them (see Fig. 2b for correspondence between panel codes and population codes). Each plot shows the mean (± SD) log probability of the data [Ln Pr (*X*|*K*)] over 10 runs (left axis, open dots and error bars) for each *K*-value. The magnitude of Δ*K* as a function of *K* determines the best-supported number of clusters in Structure analyses (right axis, black dots and continuous line).
